# The Deoptimization of Rabies Virus Matrix Protein Impacts Viral Transcription and Replication

**DOI:** 10.3390/v12010004

**Published:** 2019-12-18

**Authors:** Jun Luo, Yue Zhang, Qiong Zhang, Yuting Wu, Boyue Zhang, Meijun Mo, Qin Tian, Jing Zhao, Mingzhu Mei, Xiaofeng Guo

**Affiliations:** College of Veterinary Medicine, South China Agricultural University, Guangzhou 510642, China; lj378483477@163.com (J.L.); yuezh1009@163.com (Y.Z.); joanzh@126.com (Q.Z.); wuyuting0614@sina.com (Y.W.); zhangboyue163@163.com (B.Z.); mj_mo@outlook.com (M.M.); chintien89@163.com (Q.T.); zjwgq8912@163.com (J.Z.); meimz@126.com (M.M.)

**Keywords:** rabies virus, matrix protein, codon deoptimization, glycoprotein, replication

## Abstract

Rabies virus (RABV) matrix (M) protein plays several important roles during RABV infection. Although previous studies have assessed the functions of M through gene rearrangements, this interferes with the position of other viral proteins. In this study, we attenuated M expression through deoptimizing its codon usage based on codon pair bias in RABV. This strategy more objectively clarifies the role of M during virus infection. Codon-deoptimized M inhibited RABV replication during the early stages of infection, but enhanced viral titers at later stages. Codon-deoptimized M also inhibited genome synthesis at early stage of infection and increased the RABV transcription rates. Attenuated M through codon deoptimization enhanced RABV glycoprotein expression following RABV infection in neuronal cells, but had no influence on the cell-to-cell spread of RABV. In addition, codon-deoptimized M virus induced higher levels of apoptosis compared to the parental RABV. These results indicate that codon-deoptimized M increases glycoprotein expression, providing a foundation for further investigation of the role of M during RABV infection.

## 1. Introduction

Rabies virus (RABV) causes a fatal neurological disease in both humans and animals. More than 59,000 humans die of rabies each year, with the majority of cases occurring in developing countries. RABV is an unsegmented, negative-sense RNA virus belonging to the genus *Lyssavirus* of the family *Rhabdoviridae*. The RABV genome is approximately 12 kb in size and comprises five genes including the nucleoprotein (N), phosphoprotein (P), matrix protein (M), glycoprotein (G), and the RNA-dependent RNA polymerase (L). M is located at position 3 on the RABV genome from the 3′ -leader sequence. M contributes to RABV assembly and budding due to its interactions with both the ribonucleoprotein and G [1,2]. Finke and colleagues demonstrated that the expression of M regulates RABV genome transcription and replication [3]. Recent studies indicate that M inhibits NF-κB signaling through interactions with RelAp43 that acts to promote RABV infection [4,5,6]. The overexpression of M increases the expression of HDAC6, which increases RABV transcription and replication through microtubule depolymerization [7]. Residue 58 of M is also critical to RABV replication [8]. In previous studies, the attenuation of M expression was performed through RABV rearrangements [3,9]. However, the rearrangement of M away from the 3′-leader sequence alters the location of other genes. As such, appropriate assays that specifically alter M expression with no effects on the position of other RABV genes are required. 

Codon bias occurs in all organisms. Changing codons or codon-pair usage can alter gene expression in specific organisms. This is because each specific cell line prefers to one or two of synonymous codons during translation [10]. This method has been employed in several studies of RNA viruses [11,12,13]. Codon-deoptimized or codon-optimized G have been used to investigate RABV pathogenicity [14]. Changing the codons based on codon pair bias can attenuate viral protein expression. 

Our previous study described that RABV carrying double G (rHEP-G) expresses more G and induces higher levels of virus neutralizing antibodies compared with the parent HEP-Flury strain [15]. Now the inactivated rHEP-G strain has been licensed as a vaccine strain and used wildly in dog’s vaccination in China. In this study, the expression of M was changed through codon deoptimization or optimization to investigate the role of M during virus infection. Changed expression of M may enhance virus replication, which may decrease the inactivated vaccine cost. Therefore, we used rHEP-G strain as the parent in this study. Recombinant codon-optimized M strain could not be rescued. The codon-deoptimized M decreased virus replication during the early stages of infection and increased virus production during later stages. Codon-deoptimized M decreased intracellular genome synthesis, attenuated M expression, and increased G expression. 

## 2. Materials and Methods

### 2.1. Cells, Viruses, and Antibodies

Mouse neuroblastoma (NA) cells (Wuhan Institute of Biological Products, Wuhan, China) were cultured in RPMI 1640 (Gibco, Suzhou, China) supplemented with 10% fetal bovine serum (FBS) (Gibco, Grand Island, New York, NY, USA). Baby hamster kidney cells (BHK-21) (Wuhan Institute of Biological Products, Wuhan, China) and chicken embryo fibroblast cells UMNSAH/DF-1 (DF-1) (Chinese Academy of Sciences, Shanghai, China) were maintained in Dulbecco’s modified Eagle’s medium (DMEM) (Gibco, Suzhou, China) supplemented with 10% FBS. rHEP-G, which carries two G genes, was reported previously [15] and propagated in NA cells. Fluoresceinisothiocyanat (FITC)-conjugated anti-RABV-N antibodies were purchased from Fujirabio Inc. (Malvern, PA, USA). Anti-RABV-M and Anti-RABV-G antibodies were prepared in our laboratory [16]. Anti-RABV-N and Anti-RABV-P antibodies for Western blots analysis were purchased from Zhejiang Tongdian Biotechnology Co., LTD (Zhejiang, China). 

### 2.2. Construction of Codon-Deoptimized and Codon-Optimized M and Virus Rescue

Codon deoptimization and optimization were performed based on the mouse codon usage table available at http://www.kazusa.or.jp/codon/. Codon-deoptimized or codon-optimized M replaced the native gene in plasmid rHEP-G [15] based on the previously described strategy [16]. Recombinant RABV was rescued in BHK-21 cells as previously described [17].

### 2.3. Virus Propagation and Titration

Recombinant RABV containing codon-deoptimized M was rescued and propagated in NA cells. Virus titrations were performed through direct fluorescent antibody assays (dFA) as previously described [17]. Briefly, NA cells grown in 96-well cell-culture plates and inoculated with 10-fold serial dilutions of the indicated virus in RPMI 1640 medium at 37 °C for 2 days. The culture medium was discarded and cells were fixed with 80% acetone for 30 min at −20 °C. Cells were washed in PBS and stained with FITC-labeled anti-RABV-N antibodies for 60 min. Antigen-positive foci were counted under a fluorescence microscope (AMG, Mill Creek, WA, USA) (focus forming units per milliliter, FFU/mL).

### 2.4. Virus Growth Assays

Monolayers of NA cells cultured at 37 °C in 100 mm cell culture dishes were infected at a multiplicity of infection (MOI) of 0.01 or 3. Culture supernatants were collected at 6, 12, 24, 48, 72, 96, and 120 h post infection (hpi). Virus titers were determined in NA cells by dFA as described above. 

### 2.5. Quantitative Real-Time PCR

NA cells were infected with RABVs and cells were harvested at indicative time points. Total RNA was extracted using TRizol reagent (Magen, Guangzhou, China) according to the manufacturer’s protocol. Reverse transcription (RT) was performed using the Transcriptor First Strand cDNA Synthesis Kit (Roche, Mannheim, Germany). Each reaction was performed in triplicate using SYBR Green Master Mix (Vazyme Biotech CO., ltd., Nanjing, China). Quantitative real-time PCR (qRT-PCR) was performed in a CFX connect real-time system (Bio-Rad, Hercules, CA, USA). The levels of N mRNA, P mRNA, M mRNA, G mRNA, L mRNA, and RABV genomes (gRNA) were normalized to glyceraldehyde-3-phosphate dehydrogenase (GAPDH) as a reference gene. The transcription of viral genes was presented by mRNA/gRNA. The primers used for qPCR were described previously [16,18].

### 2.6. Western Blot

Cells infected with RABV at an MOI of 0.01 or 3 were lysed at indicated time points in RIPA buffer (Beyotian, Shanghai, China). Western blot analysis was performed as previously described [16] with antibodies against RABV-N, RABV-P, RABV-M, RABV-G, or β-actin. Protein bands were imaged on Fine-do x6 (Tanon, Shanghai, China). Gray analysis was performed using Image J software. The expression of N, P, M, or G was normalized to β-actin in NA cells. 

### 2.7. Assessment of Virus Spread

Virus spread assay was conducted using NA cells on 6-well plate as described previously [19,20]. Briefly, NA cells were infected with RABV at an MOI of 0.01, and incubated for 2 h at 37 °C. The inoculum was removed and cells were washed with PBS. Cells were covered with 1% low-melting agar containing 5% FBS. The low-melting agar was maintained at 37 °C before use. Plates were incubated at 37 °C and agar was removed at 24, 36, 48, 60 hpi. Cells were fixed in 80% acetone and stained with FITC-conjugated anti-RABV-N antibodies and DAPI. Focus forming assays were performed on a fluorescence microscope. At least 12 fluorescent foci in each well were analyzed to determine the number of infected cells per fluorescent foci.

### 2.8. Flow Cytometry

NA cells were infected with RABV at an MOI of 3 and harvested at 24 hpi. Cells were stained with the Annexin V-FITC apoptosis kit (BestBio, Shanghai, China) according to the manufacturer’s protocols. Flow cytometry was performed on a Beckman FC500 flow cytometer (Beckman Coulter, Fullerton, CA, USA). Data were analyzed using CXP Software.

### 2.9. Statistical Analysis

Data were analyzed using GraphPad Prism 6 software (GraphPad Software, San Diego, CA, USA). Statistical significance was determined using a Student’s t test. *p* < 0.05 was considered statistically significant.

## 3. Results

### 3.1. Construction of RABV-M Variants 

The codon pairs of M of the rHEP-G strain were optimized or deoptimized in *Mus musculus* based on the Codon Usage Database (http://www.kazusa.or.jp/codon/) without alterations of the amino acid sequences. Compared to the wild-type M gene, codon-optimized M contains 136 synonymous nucleotide substitutions whilst codon-deoptimized M contains 154 synonymous nucleotide substitutions (Supplementary Data). Codon-optimized and codon-deoptimized M genes were synthesized by GENEWIZ (Suzhou, China). The M gene of rHEP-G was then replaced with codon-optimized M (rHEP-G-M^max^) or codon-deoptimized M (rHEP-G-M^min^). rHEP-G-M^min^ was successfully rescued whilst rHEP-G-M^max^ could not be rescued in BHK-21 cells. rHEP-G-M^min^ was verified using immunofluorescence staining for anti-RABV-N antibodies in NA cells. The M gene of rHEP-G-M^min^ was confirmed through sequencing.

### 3.2. Codon-Deoptimized M Decreases Virus Production during the Early Stages of Virus Infection, but Increases Virus Production at Later Stages

**Figure 1:**
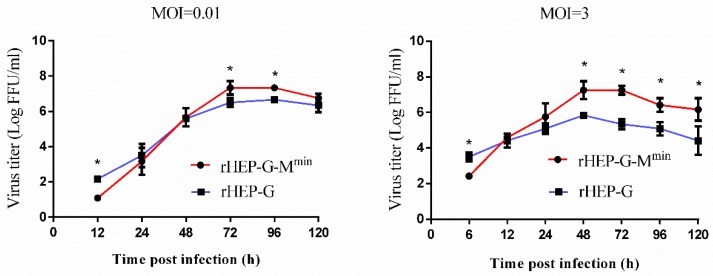
One-step growth curves of rabies virus (RABV) in mouse neuroblastoma (NA) cells. NA cells were infected with rHEP-G or rHEP-G-M^min^ at a multiplicity of infection (MOI) of 0.01 (multi-step) or 3 (one-step) at 37 °C. Culture supernatants were collected at the indicated times and virus titers were assayed in triplicate. Mean data are shown. Asterisks indicate significant differences between the groups calculated using a Student’s t test (* *p* < 0.05).

One-step and multi-step growth curves of rHEP-G-M^min^ were determined in NA cells to investigate whether the attenuated expression of M influences virus production. As shown in Figure 1, virus production in rHEP-G-M^min^ was lower than parental rHEP-G during the early stages (12 hpi at an MOI of 0.01 while 6 hpi at an MOI of 3) of infection. However, rHEP-G-M^min^ infection showed higher virus titers than the parent rHEP-G at later infection stages (72 and 96 hpi at an MOI of 0.01 while post 48 h at an MOI of 3).

### 3.3. Codon-Deoptimized M Regulates Intracellular Genomic RNA and mRNA Synthesis

**Figure 2:**
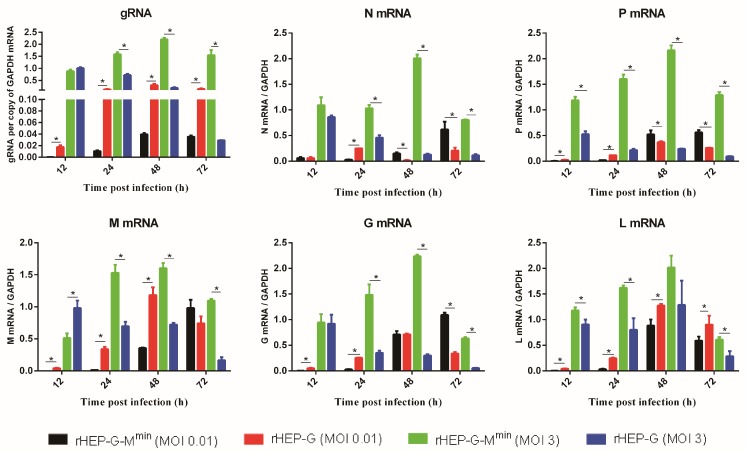
RABV genome (gRNA) and mRNA synthesis in NA cells. NA cells were infected with rHEP-G or rHEP-G-M^min^ at an MOI of 0.01 or 3 and cells were harvested at 12, 24, 48, 72 hpi. The levels of gRNA, N mRNA, P mRNA, M mRNA, G mRNA, and L mRNA in cells were determined by qRT-PCR in a CFX connect real-time system. The relative gRNA and mRNA levels were normalized to glyceraldehyde-3-phosphate dehydrogenase (GAPDH). Data represent the mean ± SD, *n* = 3. Asterisks indicate significant differences between the groups calculated using a Student’s t test (* *p* < 0.05).

We assessed the levels of genomic RNA and mRNA synthesis in NA cells following rRABV infection. As shown in Figure 2, codon-deoptimized M strain had significantly lower levels of gRNA synthesis compared to the parent rHEP-G when infected at an MOI of 0.01, but displayed significantly higher levels of gRNA synthesis compared to the parent rHEP-G when infected at an MOI of 3. For the viral mRNAs synthesis, rHEP-G-M^min^ displayed lower levels of N mRNA, P mRNA, M mRNA, G mRNA, and L mRNA at 12 and 24 hpi than rHEP-G when infected at an MOI of 0.01 while rHEP-G-M^min^ displayed higher levels of N mRNA, P mRNA, and G mRNA at 72 hpi (Figure 2). For the infection at an MOI of 3, the codon-deoptimized M strain had significantly higher levels of all the five viral gene mRNAs compared to the parent rHEP-G after 24 hpi (Figure 2).

### 3.4. Codon-Deoptimized M Regulates Transcription

**Figure 3:**
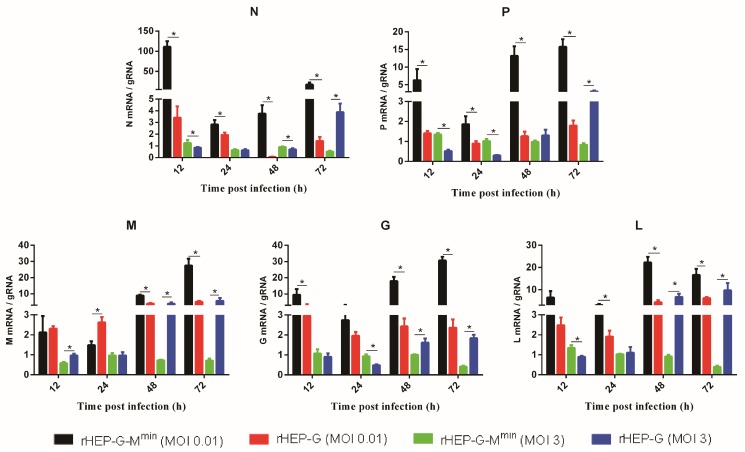
RABV mRNA/gRNA ratio in NA cells. Transcription efficiency was evaluated by using mRNA/gRNA. NA cells were infected with rHEP-G or rHEP-G-M^min^ at an MOI of 0.01 or 3 and cells were harvested at 12, 24, 48, 72 hpi. The levels of gRNA, N mRNA, P mRNA, M mRNA, G mRNA, and L mRNA in cells were determined by qRT-PCR as described in Materials and Methods. mRNA/gRNA ratio were calculated and data represent the mean ± SD, *n* = 3. Asterisks indicate significant differences between the groups calculated using a Student’s t test (* *p* < 0.05).

mRNA/gRNA ratios were investigated to valuate RABV transcription. As shown in Figure 3, rHEP-G-M^min^ displayed significantly higher levels of N, P, G, L mRNA/gRNA ratios than rHEP-G at all the time points when infected at an MOI of 0.01. For the infection at an MOI of 3, rHEP-G-M^min^ displayed higher levels of N, P, G, L mRNA/gRNA ratios at early stage (12 or 24 hpi) while rHEP-G-M^min^ displayed lower levels of N, P, G, L mRNA/gRNA ratios at 72 hpi (Figure 3). Codon-deoptimized M failed to enhance the M mRNA/gRNA ratio at early stage (12 or 24 hpi).

### 3.5. Codon-Deoptimized M Increases G Expression

**Figure 4:**
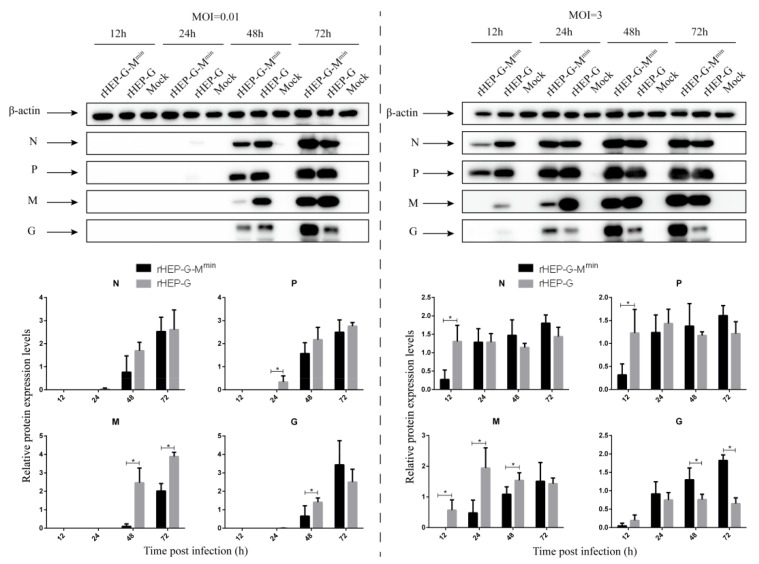
Western blot analysis of viral proteins. NA cells were infected with rHEP-dG, rHEP-dG-M^min^ at an MOI of 0.01 or 3 and cell lysates were harvested at indicated time points post-infection. Western blot analysis was used to assess the relative expression levels of N, P, M, G, and β-actin. Gray analysis was performed using Image J software. The expression of N, P, M, or G was normalized to β-actin. Data represent the mean ± SD, *n* = 3. Asterisks indicate significant differences between the groups calculated using a Student’s t test (* *p* < 0.05).

NA cells were infected with rHEP-G-M^min^ or rHEP-G at an MOI of 0.01 or 3 and the expression of other viral proteins were assessed by Western blot analysis. As shown in Figure 4, at 24 hpi, N, P, M, and G expression of rHEP-G-M^min^ were significantly lower than rHEP-G when infected at an MOI of 0.01. After 48 hpi, the expression of N and P were comparable in both rHEP-G-M^min^ and rHEP-G infected cells, but a surprising increase in G expression in rHEP-G-M^min^ compared to rHEP-G was observed at 72 hpi. For the infection at an MOI of 3, N, P, M, and G expression of rHEP-G-M^min^ were lower than rHEP-G at 12 hpi. After 24 hpi, the expression N and P were comparable in both rHEP-G-M^min^ and rHEP-G infected cells. Enhanced G expression in rHEP-G-M^min^ compared to rHEP-G was observed after 24 hpi (Figure 4).

**Figure 5:**
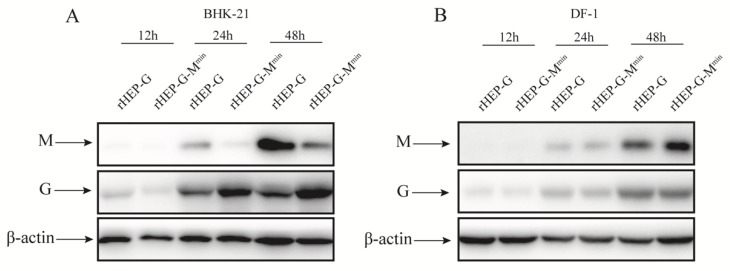
Western blot analysis of M and G proteins. BHK-21 (**A**) and DF-1 (**B**) cells were infected with rHEP-dG, rHEP-dG-M^min^ at an MOI of 3 and cell lysates were harvested at indicated time points post-infection. Western blot analysis was used to assess the relative expression levels of M, G, and β-actin.

To further confirm the findings, BHK-21 cells, which have the same codon bias compared with mouse-origin cells, were infected with rHEP-G-M^min^ or rHEP-G at an MOI of 3 and the expression of M and G were assessed by Western blot analysis. As shown in Figure 5A, codon deoptimization corresponded with decrease in expression of M and increased G expression at 24 and 48 hpi in BHK-21 cells. These data suggest that codon-deoptimized M increases the accumulation of G time dependent during virus infection.

### 3.6. Codon-Deoptimized M Fails to Increase G Expression in DF-1 Cells

In this study, M was codon deoptimized based on mouse-origin codon bias. To investigate whether the codon-deoptimized M increases G expression in the other cell line which has different codon bias compared with mouse-origin, DF-1 cells were infected with rHEP-G-M^min^ or rHEP-G at an MOI of 3 and the expression of M and G were assessed by Western blot analysis. As shown in Figure 5B, the expression of M of rHEP-G-M^min^ was comparable to rHEP-G. What is more, the expression of G of rHEP-G-M^min^ was not increased compared with rHEP-G in DF-1. This illustrate that codon deoptimization did not correspond with changes in expression of M in DF-1 cells.

### 3.7. Attenuating M Expression does not Influence RABV Spread in NA Cells

**Figure 6:**
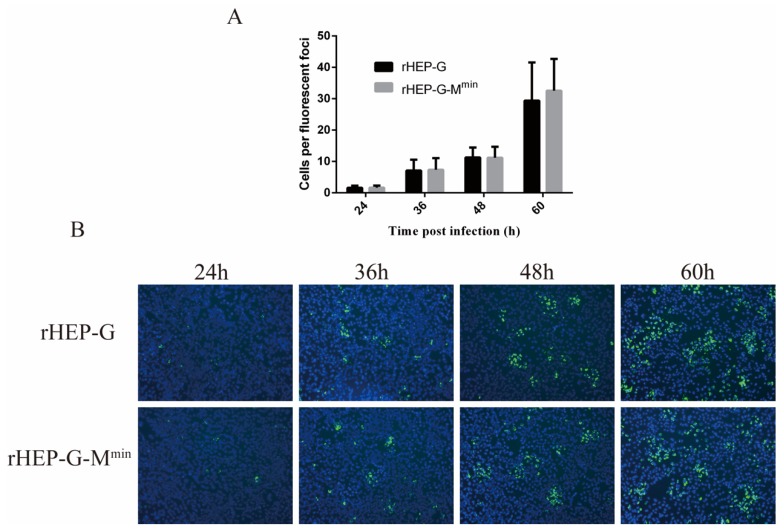
Cell-to-cell spread of RABV in NA cells. NA cells were infected with rHEP-G or rHEP-G-M^min^ at an MOI of 0.01, overlaid with 1% low-melting agar, incubated at 37 °C and cells were stained at 24, 36, 48, and 60 hpi with FITC-conjugated monoclonal antibodies against RABV-N and DAPI. Fluorescence focus forming units were determined under a fluorescence microscope (100×). At least 12 fluorescent foci in each well were analyzed to determine the number of infected cells per fluorescent foci (**A**). For each virus, at least four representative images were taken from different areas of the same well. A representative image is shown (**B**).

NA cells were infected with rHEP-G-M^min^ or rHEP-G at an MOI of 0.01 to investigate whether codon-deoptimized M affects cell-to-cell spread. As shown in Figure 6A, the cell-to-cell spread of rHEP-G-M^min^ in NA cells was similar to parental rHEP-G, suggesting no effects of attenuating M expression. Representative images were shown in Figure 6B.

### 3.8. rHEP-G-M^min^ Induces Stronger Apoptosis in NA Cells

**Figure 7:**
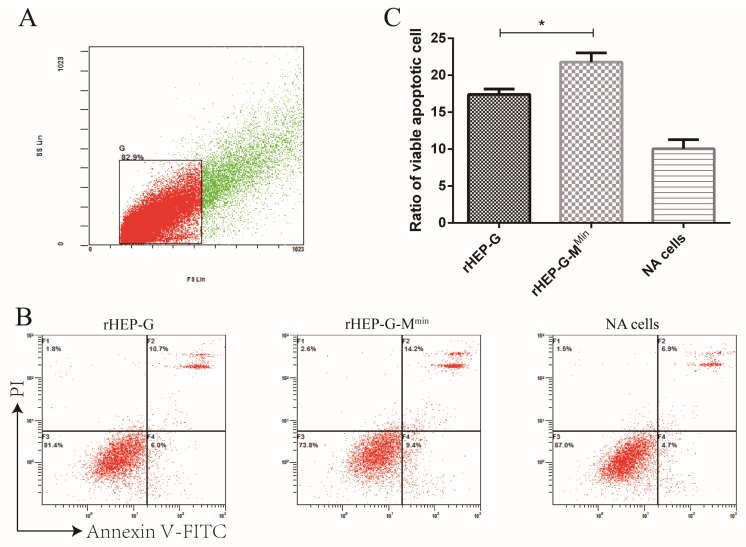
Cell apoptosis induced by RABV. NA cells were infected with rHEP-G, rHEP-G-M^min^, or mock infected at an MOI of 3 and cells were harvested by trypsin digestion at 24 hpi. Cells were stained with Annexin-V and PI for flow cytometry detection of apoptosis. Data were collected and analyzed with a Beckman FC 500 flow cytometer (Beckman Coulter) and CXP analysis software (Beckman Coulter). (**A**) Gating strategy to identify single NA cells. (**B**) Representative flow cytometry plots showing apoptotic cells in the lower right and upper right quadrants are shown. (**C**) Percentage of apoptotic cells amongst the total number of cells are shown as the mean ± SD, *n* = 4. Asterisks indicate significant differences between the two groups, calculated through a Student’s t test (* *p* < 0.05).

G induces apoptosis during RABV infection. Given the effects of M attenuation during the later stages of virus infection, we reasoned that rHEP-G-M^min^ or rHEP-G may differ in their rates of apoptotic induction. To assess this, NA cells were infected with rHEP-G-M^min^ or rHEP-G at an MOI of 3 and flow cytometry was performed following Annexin-V/PI labeling 24 hpi. A higher number of apoptotic cells were observed for rHEP-G-M^min^, compared to cells infected with rHEP-G (Figure 7). Thus, codon-deoptimized M enhances NA cell apoptosis during RABV infection.

## 4. Discussion

Our previous studies indicated that rearrangements of the M gene to position 2 or 4 from position 3 significantly decrease RABV production in vitro [9]. Another study has shown that the expression levels of M influences RABV transcription and replication [3]. In this study, to investigate the influence of M expression on virus infection, we constructed codon-deoptimized and codon-optimized M strains in which no amino acids were altered. The advantage of this system was the lack of effects on other viral proteins. The function of M in RABV infection might be more objectively investigated by this system. An interesting observation was that RABV containing a codon-optimized M gene could not be rescued. It is apparent that the overexpression of M is not beneficial to RABV recovery. In this study, the expression of M indeed decreased after M codon-deoptimization in NA cells rather than in DF-1 cells. This is because codon-deoptimization of M depends on murine cell lines. Most amino acids have different synonymous codons. Previous studies indicated that a specific organism prefers one or two of synonymous codons when they perform translation of mRNA based on different frequency of usage of transfer RNA [21,22,23]. Here, we speculate that codon-deoptimization decreased M mRNA translation, which results in reduced M protein in NA cells.

M plays an important role in the balance of RNA replication and mRNA synthesis [24]. We found that codon-deoptimized M inhibits genome synthesis during RABV infection at an early stage of infection. Additionally, the transcription ratios (mRNA/gRNA) of codon-deoptimized M strain increase. These results are consistent with the previous M attenuation study [3]. The codon-deoptimized M strain displayed lower transcription ratios at 72 hpi than the parent rHEP-G when infected at an MOI of 3. This is due to the abundant M accumulation at the late stage of infection. Unlike other viral genes, codon-deoptimized M did not enhance the M mRNA/gRNA ratio at the early stage (12 or 24 hpi). This might be due to the extremely low M expression at the early stage, which caused a transient inhibition of itself transcription. In sum, decreased M could increase RABV transcription. This enhanced-transcription effect could be amplified under a high MOI condition. Therefore, codon-deoptimized M displayed higher levels of mRNAs synthesis since 12 hpi when infected at an MOI of 3. L mRNA of rHEP-G-M^min^ increased and subsequently increased gRNA synthesis, as L plays a critical role in gRNA synthesis [25]. Therefore, we speculate that M influences the RNA replication and mRNA synthesis might be dependent on its influence on L.

Our results described that codon-deoptimized M decreases RABV titers during the early stages of infection. This might be because M expression was insufficient for RABV assembly and budding, resulting in a transient inhibition of virus production. rHEP-G-M^min^ grew more efficiently than rHEP-G in the high MOI condition rather than low MOI condition. But both MOIs showed the same growth tendency. The multi-step growth curve (low MOI) is related to the spread ability of RABV. rHEP-G-M^min^ showed the same spread ability compared to rHEP-G in NA cells at a very low MOI. This may partially result in a different growth efficiency between low MOI and high MOI. For the high MOI condition, the virus titers of rHEP-G-M^min^ were significantly higher than the parental rHEP-G strain at the later stages of infection. The higher gRNA synthesis of rHEP-G-M^min^ might account for its higher virus titers. However, for the low MOI conditions, rHEP-G-M^min^ still displayed higher virus titers than rHEP-G after 72 hpi, at which time points rHEP-G-M^min^ had a lower level of gRNA. Previous studies demonstrated that M and G play important roles in RABV assembly and budding [1,26,27]. Here, rHEP-G-M^min^ displayed equal M and more G compared to rHEP-G. Therefore, we speculate that the rate of assembly and budding of rHEP-G-M^min^ was enhanced at the later stage of infection and subsequently contributed to the higher virus titers.

Codon-deoptimized M inhibited viral protein expression during the early stages of infection due to restricted virus production. However, N and P expression in rHEP-G-M^min^ were quickly restored compared to rHEP-G, whilst the expression of G was significantly higher than rHEP-G. Thus, codon-deoptimized M enhances G expression, which has not been previously reported. N mRNA, P mRNA, and G mRNA of rHEP-G-M^min^ showed higher synthesis levels than those of rHEP-G in NA cells. However, codon-deoptimized M only increased G accumulation rather than other viral proteins. Therefore, codon-deoptimized M might also affect viral gene translation. The primary function of M is virus assembly/budding [1]. As mentioned above, codon-deoptimized M regulates RABV transcription and replication, which accord with the previous study [3]. The functions of M are much more than these. M could regulate the translation of host and viral proteins through interacting with eukaryotic translation initiation factors [28]. This also happens in vesicular stomatitis virus infection, which has been demonstrated by early studies [29,30,31]. Here, we speculate that codon-deoptimized M might decrease N and P translation and increase G translation through interaction with host proteins. G is required during virus assembly and release [24]. We thus speculate that the increased G expression promotes budding of the rHEP-G-M^min^ strain, increasing virus titers at the later stage of infection.

Previous studies described how amino acid substitutions at position 194 or 242/255/268 of G influence RABV cell-to-cell spread [20,32]. Our previous study described how the replacement of M of avirulent RABV strains with pathogenic M enhanced cell-to-cell spread in NA cells [33]. However, in this study, we found that M expression does not influence cell-to-cell spread, despite the increased G expression. In addition, G plays a critical role in the apoptotic induction induced by RABV [34]. Previous studies have shown that the enhanced levels of G lead to higher levels of apoptosis in cells infected with RABV [15,35,36,37]. In this study, we found that rHEP-G-M^min^ induces higher levels of apoptosis in NA cells. This partially was related to the enhanced G expression following the codon deoptimization of M.

In conclusion, we show that when attenuating M expression through altering codon usage, RABV replication is inhibited during the early stages of infection, but virus titers increase during the later stages. Codon-deoptimized M inhibited genome synthesis when infected at a low MOI while it increased genome synthesis when infected at a high MOI. Codon-deoptimized M enhanced transcription and G expression in NA cells. rHEP-G-M^min^ induced higher levels of apoptosis in NA cells compared to parental rHEP-G. These findings support a role for M during the regulation of RABV RNA synthesis and viral protein expression.

## Supplementary Materials

The following are available online at https://www.mdpi.com/1999-4915/12/1/4/s1.
