# Supplementary material for: The Deoptimization of Rabies Virus Matrix Protein Impacts Viral Transcription and Replication
Source: Viruses. 2019 Dec 18;12(1):4. doi: 10.3390/v12010004 (PMC7019236; doi:10.3390/v12010004)
Supplement: Supplementary file 1 [file viruses-12-00004-s001.pdf]

Supplemental data

M gene of rHEP-G (GenBank ID: AB085828.1)

ATGAACTTTCTATGTAAGATAGTGAAAAACTGTAGGGATGAGGACACCCAAAAGCCCTCTC
CCGCGTCAGCCCCTCCGGATGGCGATGACCTGTGGCTTCCACCTCCAGAATATGTCCCGCTG
AAAGAACTCACAAGCAAGAAGAACATGAGGAACTTTTGTATCAACGGGGAGGTTAAAGTG
TGCAGTCCGAACGGTTACTCATTCAGGATCCTGCGGCATATTCTGAGATCATTCGACGAGAT
ATACTCTGGGAATCATAGGATGATTGGGTTAGTCAAAGTTGTTGTTGGACTAGCTTTATCAG
GAGCTCCAGCTCCTGAGGGCATGAACTGGGTATACAAATTGAGGAGAACCCTTATTTTCCA
GTGGGCTGATTCCAGGGGCCCTCTTGAAGGGGAGGAGTTGGAACACTCTCAAGAGATCACT
TGGGACGATGATACTGAATTCGTCGGATTGCAAATGAGAGTGAGCGCAAGACAATGTCAT
ATTCAAGGCAGGATCTGGTGTATCGACATGAACTCGAGGGCATGTCAACTATGGTCTGACA
TGTCTCTTCAGACACAAAGGTCTGAAGAGGACAAAGACTCTTCTCTGCTTTTAGAATAA

Codon-optimized M

ATGAACTTCCTGTGCAAGATCGTGAAGAACTGCAGGGACGAGGACACACAGAAGCCAAGC
CCCGCCAGCGCACCACCTGACGGCGATGACCTGTGGCTGCCACCTCCAGAGTACGTGCCAC
TGAAGGAGCTGACAAGCAAGAAGAACATGAGAAACTTCTGCATCAACGGCGAGGTGAAG
GTGTGCAGCCCAAACGGCTACAGCTTCAGGATCCTGAGACACATCCTGAGATCCTTCGACG
AGATCTACTCTGGCAACCATCGGATGATTGGACTGGTGAAGGTGGTCGTGGGACTGGCCCT
GTCTGGCGCCCCAGCCCCTGAGGGCATGAACTGGGTGTACAAGCTGAGGAGAACACTGAT
CTTCCAGTGGGCCGACAGCAGAGGCCCTCTGGAGGGCGAAGAGCTGGAGCACAGCCAGGA
GATCACATGGGACGATGACACAGAGTTTGTGGGCCTGCAGATGAGAGTGTCTGCTCGGCAG
TGCCACATCCAGGGCAGAATCTGGTGCATCGACATGAACAGCAGGGCTTGCCAGCTGTGG
AGCGACATGAGCCTGCAGACACAGAGGTCTGAGGAGGACAAGGACAGCAGCCTGCTCCTG
GAGTAA

Codon-deoptimized M

ATGAATTTCTTGTGTAAGATAGTAAAGAATTGTCGTGATGAAGACACGCAAAAGCCGTCGC
CCGCATCAGCGCCGCCAGATGGTGATGACTTGTGGCTACCGCCACCGGAATATGTTCCACT
CAAAGAACTCACTTCGAAGAAGAATATGCGAAACTTTTGCATAAATGGTGAAGTCAAGGT
ATGTTCGCCAAATGGTTATTCGTTTCGGATACTACGACACATACTTCGTTCGTTTGATGAAAT
CTATTCGGGAAATCATCGAATGATAGGTCTAGTAAAGGTAGTTGTAGGGTTAGCGCTAAGT
GGAGCGCCGGCACCAGAAGGAATGAATTGGGTCTACAAATTGCGACGCACGCTAATCTTC
CAATGGGCGGATTCGCGAGGACCACTCGAAGGGGAAGAGCTAGAACATTCACAAGAAAT
AACATGGGATGACGATACGGAATTCGTTGGTCTCCAAATGAGAGTCTCGGCACGACAATGT
CACATACAAGGAAGAATATGGTGTATAGACATGAATTCACGTGCGTGTCAACTCTGGTCCG
ACATGTCGCTCCAAACGCAACGATCGGAAGAAGATAAAGATTCGTCCCTCCTTCTCGAATA
A

Alignment of the various M gene sequences

**M gene**	ATG	AAC	TTT	CTA	TGT	AAG	ATA	GTG
**Codon-optimized M**	- - -	- - -	- - C	- - G	- - C	- - -	- - C	- - -
**Codon-deoptimized M**	- - -	- - T	- - C	T-G	- - -	- - -	- - -	- - A

AAA	AAC	TGT	AGG	GAT	GAG	GAC	ACC
--G	---	--C	---	--C	---	---	--A
--G	--T	---	C-T	---	--A	---	--G
CAA	AAG	CCC	TCT	CCC	GCG	TCA	GCC
--G	---	--A	AGC	---	--C	AGC	--A
---	---	--G	--G	---	--A	---	--G
CCT	CCG	GAT	GGC	GAT	GAC	CTG	TGG
--A	--T	--C	---	---	---	---	---
--G	--A	---	--T	---	---	T--	---
CTT	CCA	CCT	CCA	GAA	TAT	GTC	CCG
--G	---	---	---	--G	--C	---	--A
--A	--G	--A	--G	---	---	--T	--A
CTG	AAA	GAA	CTC	ACA	AGC	AAG	AAG
---	--G	--G	--G	---	---	---	---
--C	--	---	---	--T	TCG	---	---
AAC	ATG	AGG	AAC	TTT	TGT	ATC	AAC
---	---	--A	---	--C	--C	---	---
--T	---	C-A	---	---	--C	--A	--T
GGG	GAG	GTT	AAA	GTG	TGC	AGT	CCG
--C	---	--G	--G	---	---	--C	--A
--T	--A	--C	--G	--A	--T	TCG	--A
AAC	GGT	TAC	TCA	TTC	AGG	ATC	CTG
---	--C	---	AGC	---	---	---	---
--T	---	--T	--G	--T	C--	--A	--A
CGG	CAT	ATT	CTG	AGA	TCA	TTC	GAC
A-A	--C	--C	---	---	--C	---	---
--A	--C	--A	--T	C-T	--G	--T	--T
GAG	ATA	TAC	TCT	GGG	AAT	CAT	AGG
---	--C	---	---	--C	--C	---	C--
--A	--C	--T	--G	--A	---	---	C-A
ATG	ATT	GGG	TTA	GTC	AAA	GTT	GTT
---	---	--A	C-G	--G	--G	--G	--C

--- --A --T C-- --A --G --A ---

GTT GGA CTA GCT TTA TCA GGA GCT
--G --- --G --C C-G --T --C --C
--A --G T-- --G C-- AGT --- --G

CCA GCT CCT GAG GGC ATG AAC TGG
--- --C --- --- --- --- --- ---
--G --A --A --A --A --- --T ---

GTA TAC AAA TTG AGG AGA ACC CTT
--G --- --G C-- --- --- --A --G
--C --- --- --- C-A C-C --G --A

ATT TTC CAG TGG GCT GAT TCC AGG
--C --- --- --- --C --C AG- --A
--C --- --A --- --G --- --G C-A

GGC CCT CTT GAA GGG GAG GAG TTG
--- --- --G --G --C --A --- C--
--A --A --C --- --- --A --- C-A

GAA CAC TCT CAA GAG ATC ACT TGG
--G --- AGC --G --- --- --A ---
--- --T --A --- --A --A --A ---

GAC GAT GAT ACT GAA TTC GTC GGA
--- --- --C --A --G --T --G --C
--T --C --- --G --- --- --T --T

TTG CAA ATG AGA GTG AGC GCA AGA
C-- --G --- --- --- TCT --T C-G
C-C --- --- --- --C TCG --- C--

CAA TGT CAT ATT CAA GGC AGG ATC
--G --C --C --C --G --- --A ---
--- --- --C --A --- --A --A --A

TGG TGT ATC GAC ATG AAC TCG AGG
--- --C --- --- --- --- AGC ---
--- --- --A --- --- --T --A C-T

GCA TGT CAA CTA TGG TCT GAC ATG

- - T   - - C   - - G   - - G   - - -   AGC   - - -   - - -
- - G   - - -   - - -   - - C   - - -   - - C   - - -   - - -

TCT   CTT   CAG   ACA   CAA   AGG   TCT   GAA
AGC   - - G   - - -   - - -   - - G   - - -   - - -   - - G
- - G   - - C   - - A   - - G   - - -   C-A   - - G   - - -

GAG   GAC   AAA   GAC   TCT   TCT   CTG   CTT
- - -   - - -   - - G   - - -   AGC   AGC   - - -   - - C
- - A   - - T   - - -   - - T   - - G   - - C   - - C   - - -

TTA   GAA   TAA
C-G   - - G   - - -
C-C   - - -   - - -
